# An Innovative Screening Panel for Preventing Miscarriages—Perspectives, Guidance and Guidelines

**DOI:** 10.3390/antiox15040464

**Published:** 2026-04-09

**Authors:** Wioleta Justyna Omeljaniuk

**Affiliations:** Department of Analysis and Bioanalysis of Medicines, Medical University of Bialystok, 15-222 Bialystok, Poland; wioleta.omeljaniuk@umb.edu.pl

**Keywords:** miscarriage, biomarkers, diagnostic potential, screening panel, prenatal care

## Abstract

**Background**: Miscarriage is the most common complication of pregnancy. Current trends in medicine point to the increasing importance of evidence-based personalization in diagnostic and therapeutic processes. **Purpose**: The aim of this study was to develop an innovative prenatal screening panel and treatment strategy for miscarriage prevention. **Results**: Previous studies have demonstrated an imbalance between oxidative and anti-oxidant mechanisms, resulting in systemic oxidative stress in women with a history of miscarriage. The importance of monitoring toxic metal concentrations as potential risk factors in early pregnancy was confirmed. The involvement of NETs in the pathogenesis of miscarriages was demonstrated, while identifying early biomarkers of this process. The effect of BPA on the activation of NETs and the development of an inflammatory response in the female participants was demonstrated. Furthermore, a mechanism of NO-dependent oxidative–anti-oxidative imbalance and NLRP3 inflammasome activation during pregnancy loss was identified in a pathway independent of NET formation, excluding apoptosis. The participation of certain microRNA molecules in reproductive failure and their value in minimally invasive diagnostics in the early stages of pregnancy have been proven. **Conclusions**: The proposed screening panel accounts for the above parameters, represents a novel approach in modern prenatal care, and prioritizes miscarriage prevention strategies.

## 1. Introduction

Miscarriage (alternative terms: spontaneous abortion or natural/early pregnancy loss) is a complication of pregnancy, involving the spontaneous expulsion of the entire fetus or parts thereof from the uterus at a stage when the fetus is not viable outside the mother’s womb [1,2]. According to the definition adopted by the WHO, a miscarriage is the termination of pregnancy before 22 weeks of pregnancy or when the fetal weight is less than 500 g [3]. Miscarriage is the most common complication of pregnancy. In Poland, an increasing trend in the incidence of miscarriages has been observed from 2000 to 2022, with a steadily decreasing number of successful pregnancies. Currently, the average annual number of lost pregnancies in Poland is 50,187, representing 8.5% of all diagnosed pregnancies. For most women, a miscarriage is a one-off event, but for some, it is recurrent. Nearly 25–30% of all women who become pregnant have experienced the loss of one or more pregnancies. It is difficult to estimate the incidence of miscarriage in the general population, as many early pregnancies are lost before they are detected. According to the available literature, miscarriage usually occurs in the first trimester of pregnancy and affects more than 80% of cases [4].

In principle, there are two types of miscarriages: those dependent on the fetus and those dependent on the mother. Among the many causes of miscarriage dependent on the mother, health determinants, including environmental, socio-economic, and occupational factors affecting the pregnant woman’s body, stand out. Poor maternal nutrition and the associated abnormal body weight (underweight, overweight, or obesity), consumption of alcohol and tobacco products, use of psychoactive substances, and dangerous diagnostic and surgical procedures are lifestyle factors that directly affect the mother’s health [5,6,7,8]. In addition, hard work involving considerable physical exertion, injuries/accidents, and high levels of stress (mental and emotional disorders) are proven causes of miscarriage. Factors present in the workplace of pregnant women include chemical compounds (medical equipment, sterilization agents, anesthetic gases) and ionizing radiation as well as the environmental pollution of the air, soil, and water (exposure to cadmium, lead, arsenic, and mercury); pesticides, plant protection products, and vehicle exhaust fumes are other serious risk factors for pregnancy loss [9,10].

Miscarriages of unknown etiologies, despite standard multifaceted diagnostics, should be considered in a broad context. The factors responsible for causing the first miscarriage may be the cause of subsequent miscarriages. Therefore, studies should be expanded to detect possible poisoning, infections (and/or inflammation), metabolic disorders (often associated with malnutrition), and endocrine and immunological disorders to evaluate this pregnancy pathology fully. Since even highly specialized and often costly tests do not provide answers to clarify the cause of all miscarriages, there is a need to search for new etiological factors in the pathogenesis of early pregnancy loss [3,5].

**Figure 1:**
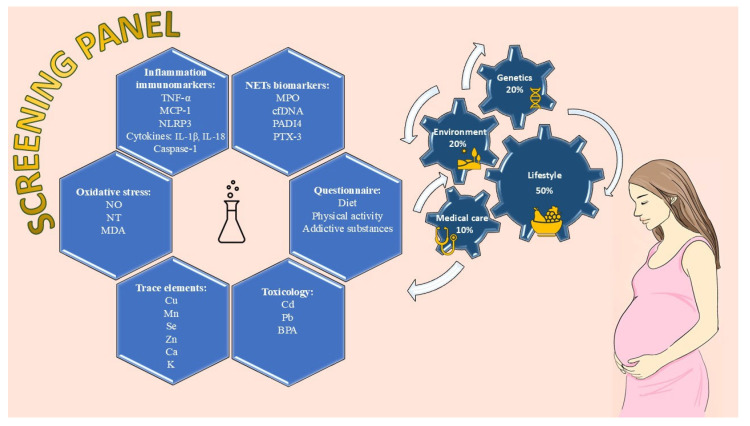
The new panel of primary screening tests for prenatal care prevention.

This study summarizes information on several new biomarkers associated with miscarriages, which should be included in the basic screening panel as part of preventive prenatal care (Figure 1).

**Figure 2:**
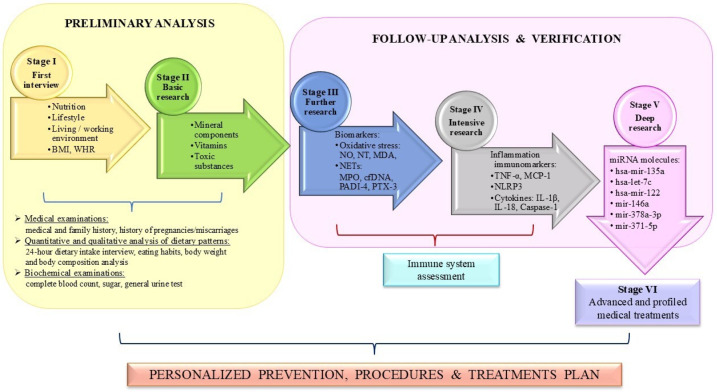
The personalized prevention, procedure and treatment plan.

The comprehensive integration and synthesis of these data would facilitate an individualized, patient-centered approach at each antenatal consultation with the clinician overseeing the pregnancy. A stepwise management algorithm is proposed, outlining potential therapeutic modifications or minor adjustments during the preconception period and throughout the pregnancy (Figure 2):

Preliminary analysis:

Stage I: The collection of the medical history, including the current health status and obstetric history. An assessment of nutritional status using validated methods (e.g., 24 h dietary recall and dietary questionnaires evaluating eating habits) as well as lifestyle factors (physical activity and leisure time activities). An evaluation of occupational and residential environmental risk factors, including the type of work and workplace characteristics. A body composition analysis based on anthropometric measurements (BMI, WHR, body fat percentage, and muscle mass).

Stage II: The performance of diagnostic analyses of biological samples (blood, hair, and urine), including an assessment of vitamin levels, anti-oxidant status (e.g., GSH-Px, TAS), essential elements (Se, Zn, Cu, Mn, Ca, K), and toxic substances (e.g., BPA, Cd, Pb).

Follow-up analysis and verification:

Stage III: An analysis of the biomarkers of oxidative stress (e.g., NO, NT, MDA) and NET formation (e.g., MPO, cfDNA, PADI4, PTX-3).

Stage IV: An analysis of inflammatory biomarkers, including TNF-α, MCP-1, NLRP3, and cytokines (IL-1β, IL-18, and caspase-1).

Stage V: An analysis of selected microRNAs (e.g., hsa-miR-135a, hsa-let-7c, hsa-miR-122, hsa-miR-146a, hsa-miR-378a-3p, and hsa-miR-371-5p).

Stage VI: The verification and comprehensive interpretation of patient results. The monitoring and implementation of personalized, targeted therapeutic interventions in accordance with clinical recommendations and guidelines provided by the gynecologist/obstetrician.

## 2. The Role of Anti-Oxidants in the Pathogenesis of Miscarriage

During a healthy pregnancy, there is a balance between pro-oxidative factors, such as free radicals, and anti-oxidant systems. These systems include enzymes containing elements such as selenium (Se), zinc (Zn), manganese (Mn) and copper (Cu), which form the first line of defense against free radicals. There are two types of anti-oxidants in the human body: enzymatic anti-oxidants (including superoxide dismutase, catalase, glutathione peroxidase, and glutathione reductase) and non-enzymatic anti-oxidants, which are affected by diet (vitamin C, vitamin E, Se, Zn, Mn, Cu, taurine, hypotaurine, glutathione, β-carotene, and carotene) [3,4]. A significant increase in systemic markers of redox imbalance accompanies advanced and irreversible stages of miscarriage, which does not occur in mild pregnancy complications. There are numerous publications on the impact of oxidative stress on female reproduction, including the pathophysiology of miscarriages [6,7].

Deficiencies of certain trace elements (Se, Zn, Cu, and Mn) may cause adverse pregnancy outcomes, including fetal growth restriction, pre-eclampsia, and an increased risk of lifestyle diseases in adulthood [8,9,10].

The results of Omeljaniuk et al.’s study showed that the total anti-oxidant status (TAS) was significantly lower in women who had experienced pregnancy loss compared to women who had a successful pregnancy. The study of anti-oxidant elements revealed that a reduced Cu concentration and an increased Mn concentration in the biological material tested differentiated women who experienced miscarriage from the control group [11].

These results indicated that women who had a miscarriage experienced a shift in the balance between the efficiency of the anti-oxidant defense system and the action of pro-oxidative factors, leading to a systemic redox imbalance, which can pose a serious threat to the life and healthy development of the fetus [11].

## 3. The Role of Cadmium (Cd) and Lead (Pb) in Miscarriage

Rapid industrial development has contributed significantly to environmental pollution by heavy metals. The risks associated with heavy metals arise directly from their transfer through the food chain—contaminated soil ⟶ plants ⟶ animals ⟶ humans—and their accumulation in the final link [12]. Cadmium and lead are toxic elements that, when ingested in larger quantities through food and drinking water, accumulate in specific organs, including the blood, liver, kidneys, brain, bones, and placenta [13,14]. The impact of heavy metals is not immediate; it emerges after years of exposure and accumulation in the body, even across generations. The accumulation of heavy metals in the body begins in prenatal life, due to the crossing of the placental barrier in women exposed to them. A child’s body is susceptible to heavy metals due to the immaturity of its systems and organs and thus the impairment of its toxin excretion mechanisms.

Recent studies have shown alarmingly higher levels of cadmium and lead in women than in men. It is noteworthy that exposure to these elements during pregnancy harms not only the woman but also the development of the placenta and, consequently, the fetus. Unfortunately, the placenta does not provide an effective barrier for the transfer of heavy metals, including cadmium and lead. After penetrating the placenta, toxic elements accumulate in the systems and organs of the fetus, disrupting their normal functioning and ultimately leading to its death [13,14]. Studies by other authors have shown abnormalities in the placental structure and function, endocrine failure, nutrient transport disorders, low birth weights, fetal syndromes, and congenital malformations, as well as the rupture of the amniotic sac and preterm delivery in women exposed to heavy metals contained in tobacco smoke. It is believed that cadmium, which partially crosses the placental barrier, harms prenatal brain development, increasing the risk of ADHD and autism. In turn, the placenta does not provide a barrier for Pb, and children fed with breast milk from exposed mothers receive further doses of lead compounds through their diet [15,16,17,18]. Multiple studies by various authors, including Omeljaniuk et al., offer detailed insights into the mechanisms and extent of the heavy metal transfer from the mother to the fetus via the placenta. These studies also emphasize the significant adverse effects of heavy metals on prenatal development and the increased risk of complications, including pregnancy loss [15,16,17,18,19].

A key finding of Omeljaniuk et al.’s project was to confirm the significant role of cadmium and lead in miscarriages [19]. These findings showed significantly higher average levels of Cd and Pb in both blood and placental tissue in women who experienced miscarriages compared to the control group. This observation revealed a relationship between the metals studied in terms of placental accumulation, which could negatively impact the pregnancy’s course and the baby’s health [19].

Omeljaniuk et al. analyzed the molar ratio between the concentrations of the tested heavy metals and several anti-oxidant elements (determined as part of another project [11]) in the blood of both study groups of women. This finding can be a useful prognostic factor in the groups of female patients. Such an analysis is particularly important in women with concomitant inflammatory diseases, especially when it concerns anti-oxidant elements in individuals with a severe selenium deficiency, as we have demonstrated in previous studies [11].

As a management priority to reduce the risk of miscarriage, there appears to be a need for effective interventions in society that aim to reduce or eliminate the smoking of tobacco products. Pregnancy may provide the strongest impetus to quit this habit. Raising awareness of the harmful effects of tobacco smoke has the potential to reduce smoking among pregnant and breastfeeding women and/or to identify the need for possible nicotine replacement therapy for those who are unable to quit.

The implementation of appropriate prevention strategies during the prenatal period by promoting health-seeking attitudes and nutritional education targeting women of reproductive age appears to be a key challenge for various health services and organizations. An important finding of Omeljaniuk et al.’s research was the observation of a correlation between toxic elements and anti-oxidant elements, which provides a strong justification for the expansion of the screening panel used in prenatal care to include these parameters [19].

## 4. The Role of Extracellular Neutrophil Traps (NETs) in Miscarriage

The causes of miscarriage are numerous and can be classified as systemic, environmental, genetic, hormonal, anatomical, infectious, and immunological. Since heavy metals and other disruptors found in the human environment can disrupt the functioning of the immune system, we assessed non-specific, innate, and inherited defense mechanisms in the course of a miscarriage, as a continuation of Omeljaniuk et al.’s previous project.

The redox balance in a woman’s body is crucial for the proper development of a pregnancy, especially in its early stages. Oxidative stress is a state of imbalance between the production of reactive oxygen species (ROS) and the ability of the body’s natural defense mechanisms (anti-oxidants) to remove them and protect against their effects. In women with ongoing miscarriages, it causes uncontrolled changes, leading to a number of disorders due to permanent cell damage, as well as endothelial dysfunction within the trophoblast [20,21,22,23]. The adverse effect of ROS on the trophoblast vascular endothelium may cause a profound disruption of its function in transporting molecules from the maternal circulation to the embryo and fetus, as well as in intercellular signaling [20,24,25].

The source of ROS may be neutrophils, which contain NADPH oxidase that catalyzes reactions, leading to the formation of oxygen radicals (respiratory burst), creating an antibacterial defense mechanism for the body [26]. Pro-oxidants may also be produced in neutrophils as a result of the activity of nitric oxide synthases (NOSs) responsible for the synthesis of nitric oxide (NO) [27]. First described in 2004, extracellular neutrophil traps (NETs), formed with the participation of NADPH oxidase, may contribute to an increase in local ROS levels. The primary function initially discovered for NETs is to defend the body against pathogens. However, recent data indicate a negative effect of excessive numbers or excessive durations of NETs. A significantly higher quantity of NETs was observed in the intervillous space of the placenta in patients with pre-eclampsia than in healthy pregnancies. It has been noted that the release of NETs consisting of DNA strands, granule proteins, and histones by neutrophils has causes ROS to be present in the extracellular space [28,29].

The most significant finding of Omeljaniuk et al.’s research was the demonstration of the extracellular presence of elementary components of extracellular neutrophil traps, such as myeloperoxidase (MPO) and histones (H2A, H2B, and H3), in placental tissue. High concentrations of NET marker proteins, including MPO and pentraxin 3 (PTX-3), were also observed in the serum of women who experienced miscarriage. These results provide strong evidence for the involvement of NETs in the premature termination of pregnancy [30]. The key to analyzing the results of this research was to identify two groups of patients who had miscarriages based on changes observed in the placental tissue. The first group consisted of patients with a history of miscarriage who did not have NET structures in placental tissue samples, and we designated them as “NET-negative”. The second group was identified among miscarriage patients with NET structures present in the placenta and termed “NET-positive”.

A highly valuable finding in this study was the observation of the highest concentrations of pro-inflammatory MPO and protective PTX-3 in serum, accompanying the presence of NETs in the placenta of miscarriage patients (“NET-positive”). This allowed these factors to be identified as early biomarkers of miscarriage. Their presence is most likely associated with the formation of NETs. PTX-3 has been identified as an important regulator of normal pregnancy and a marker of inflammation in pre-eclampsia, primarily through its ability to bind histones and mitigate their cytotoxic effects on endothelial cells. In turn, the positive correlation between MPO and PADI4 enzymes in “NET-positive” patients confirmed the participation of this enzyme in the formation of NETs in pregnant women. The literature indicates that a deficiency of this enzyme correlates with reduced NET production and, in turn, reduced inflammation, which protects against miscarriage. Therefore, the decrease in the PADI4 enzyme concentration observed in women who miscarried, together with the increase in the PTX-3 concentration, may suggest the presence of a regulatory and/or protective mechanism that limits NET formation during pregnancy.

The negative correlation between PADI4 and nitrotyrosine (NT), which correlated positively with malondialdehyde (MDA) in the control group, indicated an intensification of pro-oxidative processes during pregnancy via a pathway other than ROS/NETs. The observed sharp increase in NO and NT levels, and the positive correlation between MDA and NT in “NET-negative” patients, indicated an enhanced pro-oxidant state and its effects in this group of women. In contrast, the low NO concentrations and changes in NT levels in “NET-positive” women allowed us to speculate that the oxidant–anti-oxidant imbalance was most likely due to an excessive increase in free radical levels accompanying NETs [30].

Excessive neutrophil activation and NET levels may increase the risk of pre-eclampsia, which poses a threat to the life of the fetus and mother. The disruption of the production and/or prolonged presence of NETs may lead to the formation of anti-neutrophil antibodies and antibodies that work against the patient’s own DNA, resulting in autoimmunity [28,31].

Data from Omeljaniuk et al.’s extensive study allowed us to identify two slightly different mechanisms of miscarriage in the study groups, related to NET formation and the oxidant–anti-oxidant imbalance. The results in “NET-positive” patients suggested that the miscarriage may have been due to the direct adverse effects of NET components. In contrast, the results in “NET-negative” patients stemmed from oxidative stress and may play a role in miscarriage. Several new aspects of NET generation have been clarified, but only a clear understanding of this mechanism will enable this knowledge to be used to prevent pregnancy loss in the future [30].

The studies conducted by Omeljaniuk et al. suggest that immunological biomarkers of NET formation and the inflammatory process may be of great diagnostic and prognostic value for assessing the risk of miscarriage. Undoubtedly, increasing the size of the participant group and conducting large-scale clinical trials would be essential to obtain reliable clinical evidence.

## 5. The Link Between Xenoestrogen Bisphenol A (BPA) and Miscarriage

The problem of environmental contamination is not limited to heavy metals; endocrine-disrupting chemicals (EDCs) with high endocrine toxicity potential are gaining prominence. The US Environmental Protection Agency defines EDCs as exogenous agents that interfere with the synthesis, secretion, transport, metabolism, and elimination of hormones that regulate homeostasis, reproduction, and development [32,33,34,35,36,37]. The WHO defines EDCs as exogenous substances or mixtures that alter endocrine system functions, causing adverse effects in humans, their offspring, or subpopulations [34]. The effects of environmental exposure to EDCs are of medical concern, particularly in the context of the potentially harmful effects on fetal development. Bisphenol A (BPA) is an EDC that can interact with the endocrine and immune systems, with effects that can persist for years after exposure or accumulation. The main route of human exposure to BPA is through the consumption of plastic-packaged food and water. The increasing presence of this xenoestrogen in many everyday items—from food containers to toys, medical products, cosmetics, and others—is worrying and correlates with hormonal disorders and, consequently, reproductive disorders [28,30]. Although the reproductive toxicity of BPA has been proven, its exact mechanisms of action are still unknown.

Due to the multifactorial etiology of miscarriage, a potential trigger is sought for genetic, immunological, endocrinological, morphological and anatomical, infectious, or iatrogenic changes that characterize this type of obstetric failure [1,2,3].

A highlight of the work by Omeljaniuk et al. was the demonstration of the role of BPA in pregnancy loss through the formation of NETs accompanied by inflammation [37]. BPA can interact with the endocrine system and modulate its function in a manner similar to estrogen, by binding to membrane and/or nuclear estrogen receptors, thereby exerting a negative impact on health, especially on reproductive and fetal development processes [32,33,34,35,36].

This conclusion was further confirmed by the positive correlation between BPA and nicotinamide adenine dinucleotide phosphate oxidase 1 (NOX1) in miscarriage patients, proving the existence of a relationship between high bisphenol concentrations and the increased activation of the NADPH oxidase complex, and may indicate a mechanism of pregnancy loss associated with the ROS/NET pathway. In turn, the highest concentrations of tumor necrosis factor α (TNF-α) and monocyte chemoattractant protein-1 (MCP-1), pro-inflammatory proteins synthesized by neutrophils with an autocrine effect, were observed in the serum of miscarriage patients in the “NET-positive” group, supporting the view that active inflammation accompanies NETs. Furthermore, the positive correlation between BPA and MCP-1 confirmed the development of inflammation associated with the accumulation of this xenoestrogen in the studied patients [37].

The results of Omeljaniuk et al.’s research indicate the high diagnostic potential of immunological biomarkers of NET formation and inflammation in assessing the predisposition to miscarriage. However, the greatest difficulty associated with estimating the dangerous level of BPA is the non-linear response of the body to BPA and individual variability, which is why couples undergoing infertility treatment or women with recurrent miscarriages should pay particular attention to limiting their exposure to BPA [37].

## 6. Determination of NLRP3 Inflammasome Potential in Miscarriage

Immune disorders are an increasingly common cause of problems related to conceiving, which, according to estimates, already affect approximately 10–15% of infertile couples [1,2,3,4,5,6,7,8,38]. Recent studies have shown a correlation between pro-inflammatory cytokine activity and pregnancy loss. Triggers and co-activators of signaling pathways leading to inflammation are mainly infections and placental inflammation [39]. According to Grewal et al. and Giakoumelou et al., an abnormal vaginal microbiome or fetal karyotype may be associated with miscarriage through inflammation [39,40].

Inflammasomes are intracellular protein complexes that initiate inflammatory responses to bacterial, viral, and fungal infections, as well as to cellular stress and tissue damage [39,40,41,42,43,44]. Their primary function is to regulate the secretion of the pro-inflammatory cytokines IL-1β and IL-18 by activating caspase-1 [45,46,47]. The result of the caspase-1 activity that leads to pore formation in the membrane is a loss of the membrane ionic gradient and an increase in osmotic pressure due to water influx, leading to cell swelling and osmotic lysis and ultimately cell death by pyroptosis, a morphologically distinct form of cell death from apoptosis [39,43]. In recent years, there has been a significant expansion of knowledge about the mechanisms of inflammasome activation, their structure, and their roles in various pathological conditions [42,45,48]. It has been shown that the NLRP3 inflammasome signaling pathway affects endometrial receptivity, i.e., the readiness of the uterine lining to accept and implant an embryo, and embryo invasion by inducing epithelial–mesenchymal transitions. Thus, the abnormal activation of inflammasomes in the endometrium may adversely affect endometrial receptivity. The excessive activation of the NLRP3 inflammasome mediates an impaired maternal–fetal inflammatory response and may be associated with complications of pregnancy, including pre-eclampsia, preterm labor, or miscarriage [46,49,50,51,52]. The latest research findings prove the existence of a sterile inflammation mechanism based on the interaction of NETs with the NLRP3 inflammasome [53].

Omeljaniuk et al.’s studies have demonstrated the involvement of the NLRP3 inflammasome in miscarriage through a pathway independent of NET formation, excluding apoptosis [53].

The highest diagnostic specificity achieved in studies presented by Omeljaniuk et al. for NLRP3 and IL-18, the highest test power for IL-18, and the significant diagnostic power of this cytokine test relative to the cut-off value indicated their potential as new biomarkers of miscarriage [53].

The direct involvement of apoptosis in pregnancy loss in the group of miscarriage patients in Omeljaniuk et al.’s study was ruled out by low FasL protein expression and the fact that there were no changes in the Fas receptor expression in placental tissue, as well as no changes in the cytochrome C concentration in the serum of these patients. Cytochrome C is released during caspase-independent apoptosis, in which calpain acts autonomously, activated by Ca ions following a stimulus (e.g., oxidant–anti-oxidant imbalance or infection). Apoptosis also appears to be ruled out in this study by both the low Ca ion concentrations in blood and the negligible presence of Ca deposits in the placental tissue of miscarriage patients. Evaluating other pro-apoptotic parameters in a large cohort of pregnant women and women planning a pregnancy, excluding those with comorbidities, could confirm or reject the proposed hypothesis. An extremely valuable finding of the statistical analysis was the highest diagnostic sensitivity for Ca, the highest positive predictive value, and the significant diagnostic power relative to AUC, suggesting that this element may be helpful for the early diagnosis of miscarriage risk. A common feature of all miscarriage patients and women in the “NET-negative” group was a significantly reduced level of Ca and K in whole blood.

The results of Omeljaniuk et al.’s previous studies [11,30,37] and those obtained in the NLRP3 project [53] indicated the activation of the oxidative stress–inflammasome pathway during miscarriage in women from the “NET-negative” group.

These results demonstrate that prevention, diagnosis, and treatment targeting the NLRP3 inflammasome may offer a breakthrough in preventing miscarriages in the near future. The inclusion of the above parameters (NLRP3, cytokines IL-1β and IL-18, caspase-1, Ca, and K ions) in the screening panel may set trends in modern prenatal care and the priority direction of miscarriage prevention strategies, especially in the face of a deepening demographic decline and a dramatically increasing number of reproductive failures.

## 7. The Role of miRNA Molecules in the Etiology of Miscarriage—Current Knowledge and Research Perspectives

A new direction in modern diagnostics is the increasingly widespread use of microRNA (miRNA) expression analysis. MicroRNAs are short, non-coding, single-stranded RNA molecules that, in most cases, act as regulators of gene expression at the post-transcriptional level, controlling the proper execution of cellular processes and ultimately affecting the levels of individual proteins in the body. MicroRNA molecules are engaged in many processes that are crucial for the development and function of the body, such as cell division and differentiation. They are also associated with the stress response, tissue remodeling, inflammation and disease states, including pregnancy pathologies such as pre-eclampsia, premature birth, macrosomia or low birth weights, and even pregnancy loss [54,55,56,57,58]. Based on the results of other researchers, the expression of candidate microRNA molecules (e.g., hsa-mir-135a, hsa-let-7c, hsa-mir-122, mir-146a and mir-378a-3p, mir-371-5p) can be used as a minimally invasive diagnostic biomarker as early as the first weeks of pregnancy. These biomarkers could be used to monitor the personalized clinical care of women in early pregnancy, particularly after a first miscarriage [59].

A significant aspect of research of this kind is the need to standardize methodologies, both at pre-analytical and subsequent analytical levels, involving large cohorts of women who have experienced miscarriage, pregnant women, and women who have had successful pregnancies, as well as the proper biobanking of samples.

Rapidly developing medical laboratory diagnostics are embracing modern technologies and setting trends for a multifaceted diagnostic approach. The development of a novel screening panel for women planning to become pregnant or in early pregnancy will enable more effective coordinated care for pregnant patients and, in the future, contribute to reducing the miscarriage rate in Poland.

## 8. Study Strengths and Limitations

**Strengths of this study:**

1. The application of an innovative, multidirectional, and comprehensive approach to miscarriage, encompassing clinical, diagnostic, and pathophysiological aspects.
2. A systematic synthesis and clear presentation of practical recommendations supporting coordinated medical care for pregnant women and those planning pregnancy, incorporating an interdisciplinary model of management.
3. The precise delineation of potential cause-and-effect pathways and stages of diagnostic and therapeutic management in cases of pregnancy loss, as well as in the assessment of women preparing for conception.
4. The use of selected biochemical parameters measured with widely available, cost-effective, and well-established methods in routine clinical diagnostics, enhancing the feasibility of implementing the proposed solutions in clinical practice.

**Limitations of this study:**

1. The limited willingness of participants—both in the study and control groups—to engage in pilot studies, likely due to heterogeneous emotional states and limited awareness of the purpose and rationale of the proposed diagnostic procedures.
2. Small sample sizes in both the study and control groups, along with a lack of validation in large, independent cohorts, limiting the generalizability of the findings.
3. The restricted scope of diagnostic procedures due to insufficient research funding.
4. The available results include both observational and experimental data.

## 9. Conclusions

The growing problem of miscarriages, which can trigger recurrent miscarriages, should be analyzed not only from an individual perspective but also from the perspective of the entire population, especially in countries with low fertility rates.

The main aim of this research was to provide primary research evidence to inform the development of a new panel of primary screening tests for prenatal care prevention (Figure 1). The proposed management algorithm is structured as a sequence of stepwise stages and constitutes a structured decision-making tool that enables the identification and implementation of potential therapeutic modifications or minor corrective interventions. It can be applied both in the preconception period and throughout pregnancy, taking into account factors that may increase the risk of miscarriage (Figure 2).

## Data Availability

The original contributions presented in this study are included in the articles. Further inquiries can be directed to the corresponding author.
